# Cognitive insight is associated with perceived body weight in overweight and obese adults

**DOI:** 10.1186/s12889-021-10559-5

**Published:** 2021-03-19

**Authors:** Sharain Suliman, Leigh L. van den Heuvel, Sanja Kilian, Erine Bröcker, Laila Asmal, Robin Emsley, Soraya Seedat

**Affiliations:** Department of Psychiatry & MRC Genomics of Brain Disorders Unit, Stellenbosch University, PO Box 241, Cape Town, 8000 South Africa

**Keywords:** Cognitive insight, Cognitive function, Neurocognition, Weight perception

## Abstract

**Background:**

Accurate perception of body weight is necessary for individuals with a high body mass index (BMI) to initiate strategies to improve their health status. Furthermore, identifying factors that influence accurate body weight perception can assist in designing appropriate educational and weight management programs. We therefore aimed to investigate whether levels of cognitive functioning and insight influence the ability to correctly judge body weight.

**Methods:**

One hundred and eighty four overweight and obese adults who participated in a cross- sectional case-control study and were controls in the aforementioned study were included. The study was conducted in Cape Town, South Africa. Demographic, weight-related, neuropsychiatric, neurocognitive and cognitive insight measures were administered. Regression analysis was conducted to determine the factors associated with correct weight perception.

**Results:**

The final regression model explained 52.3% of variation in accurate perception of body weight and was significant (*p* ≤ 0. 001). The model correctly classified 79.3% of individuals who were able to correctly and incorrectly judge their weight. Adults with higher BMI, and lower self-certainty, those who reported that they had gained weight in the previous year and those who were told by a healthcare professional to lose or maintain a healthy weight were more likely to correctly judge their weight.

**Conclusion:**

Some aspects of cognitive insight (self-certainty) but not cognitive functioning were associated with perception of body weight in this sample. Awareness of recent weight changes, higher BMI and advice from of health care professionals were also significantly associated with perception of body weight, while demographic variables were not. Understanding the factors that contribute to the correct perception of weight is important in identifying appropriate health interventions that may address the burden of associated non-communicable diseases in overweight and obese individuals.

**Supplementary Information:**

The online version contains supplementary material available at 10.1186/s12889-021-10559-5.

## Background

Overweight and obesity are risk factors for numerous health problems, including cardiovascular disease, diabetes, hypertension, high cholesterol, musculoskeletal disorders and some forms of cancer [1, 2]. Obesity is associated with a higher risk of chronic illness and consequently significant additional health care costs [1, 2]. Increasing rates of obesity are, therefore, a public health concern worldwide, and particularly in many low- and middle-income countries (LMIC) where, in addition to the ongoing problems of infectious diseases and undernutrition, there is also a rapid increase in non-communicable disease risk factors such as overweight and obesity [2]. This has been referred to as the ‘double burden of malnutrition’ [3].

Perceived body weight (PBW) is the personal evaluation of one’s weight as “underweight”, “normal weight” or “overweight” irrespective of actual Body Mass Index (BMI) [4–6]. Some individuals incorrectly judge their own body weight, with overweight individuals tending to underestimate their body size and underweight individuals tending to overestimate their body size. In the South African National Health and Nutrition Examination Survey, 84.5% of participants had a distorted perception of their weight [7].

Several studies have documented an association between incorrect perception of weight and mental health problems. Cross-sectional studies have reported associations with psychological distress, depression, and suicidal ideation, and even work absenteeism [8–12]. A longitudinal study conducted in Australia similarly found that the perception of being overweight during adolescence was a significant risk factor for later depression in young adult men and women [13]. In contrast, other studies have reported protective effects of weight misperception. A longitudinal study found that weight misperception among overweight/obese adolescents of white ethnicity in the USA may be protective against depression in adulthood [14]. Other cross-sectional studies have also found a protective effect of misperceived weight on mental health [15, 16].

Accurate perception of body weight is influenced by a number of factors, many of which affect actual weight, including age, gender, family, societal values, ethnicity, education level and socio-economic status (SES) [4, 17–21]. Cognitive functioning, particularly executive functioning, has also been linked to excess weight [22–25] as well as to accurate perception of body weight [26]. Cognitive insight, a process of self-evaluation that considers both an individual’s ability and willingness to objectively reflect on their beliefs, and their certainty that these beliefs are correct, is believed to be associated with cognitive functioning [27–29]. Previous studies suggest that poor cognitive insight is associated with impaired episodic memory [30] and executive functioning [31]. PBW may thus be influenced by cognitive functioning and insight.

To our knowledge, there has been no study evaluating the relative contributions of cognitive functions and cognitive insight on PBW. Identifying factors that influence or predict poor body weight perception can assist in designing more appropriate educational and weight management programs. In this study we investigated PBW with a particular focus on underestimating weight in a cohort of South African adults to determine the prevalence as well as the cognitive and socio-demographic correlates of accurate perception of weight. We further investigated the association between cognitive performance and insight and the ability to correctly judge body weight. Specifically, we asked “Does cognitive functioning influence PBW?”, “Does cognitive insight play a role in PBW?”, and “Which socio-demographic features influence PBW?” Individuals with abnormal weight who do not accurately perceive their own body weight may be less likely to initiate strategies to improve their health status, further highlighting the need to better understand the determinants of overweight/ underweight in LMICs and in ethnically diverse populations [17, 18]. This research adds to the scarce literature on the relationship between cognition and weight perception, particularly in LMICs and non-Caucasian populations.

We hypothesised that (i) better cognitive functioning would be associated with improved accuracy of PBW; (ii) better cognitive insight (higher self-reflectiveness, i.e. objectivity and reflectiveness) and lower self-certainty (i.e. overconfidence in own beliefs) would be associated with correct PBW; and (iii) socio-demographic factors such as age, gender, education level, and income would influence PBW.

## Methods

### Participants

Participants were selected from *N* = 314 mixed-race adults (aged ≥18 years), recruited as non-psychiatric controls for a cross-sectional, case-control project titled “Understanding the SHARED ROOTS of Neuropsychiatric Disorders and Modifiable Risk Factors for Cardiovascular Disease study”. Participants were recruited through community newspaper advertisements and fliers. The study was approved by the Stellenbosch University Faculty of Medicine and Health Sciences Human Research Ethics Committee (reference number: N13/08/115), conducted in accordance with the Declaration of Helsinki, and all participants signed informed consent. Participants were excluded if they were unable to read, write and understand the Informed Consent documents in English or Afrikaans, diagnosed with a major medical illness (e.g., epilepsy, cancer, HIV, stroke), or psychiatric disorder (e.g. schizophrenia, other psychotic disorder, bipolar disorder, substance use disorder, anxiety or depressive disorder) or neurological disorder, had experienced a head injury with loss of consciousness, were on psychiatric medication, or pregnant.

### Measures

Neuropsychiatric and neurocognitive measures were administered by clinicians and researchers trained in their use and in the participants’ language of choice (English or Afrikaans). Appropriate cultural and language adaptations were made to the neurocognitive tests and test instructions with stimuli and response booklets translated into Afrikaans and back-translated into English by independent translators [32, 33] (Personal communication with Dr. Randolph, 2014). Reliability (Cronbach’s alpha) was in the acceptable range (0.7–0.8) for all instruments.

- A demographic questionnaire included sociodemographic factors (age, gender, years of education, income, marital status), and past and current medical and psychiatric history.
- The MINI International Neuropsychiatric Interview (MINI- version 6) was used to assess for major psychiatric disorders, including Major Depressive Episode. The MINI has been shown to have high validity and reliability scores when compared with results from the Structured Clinical Interview for DSM-III-R, the Composite International Diagnostic Interview, the Diagnostic Interview Schedule and the Present Status Examination [34]. Participants who screened positive for any disorder were excluded from the analysis.
- Height (cm) and body weight (kg) were objectively measured by study nurses. Weight status was subsequently determined according to standardized body mass index (BMI) classification: < 18.5 – underweight; 18.5–24.9 – normal; 25–29.9 – overweight; 30–34.9 – obese class I; 35.0–39.9 – obese class II; > 40.0 – obese class III [35].
- Current weight perception was based on a self-report question –where individuals were asked to estimate their own weight according to three categories (underweight, normal weight, overweight). Individuals were stratified according to whether they correctly or incorrectly perceived their current body weight status and whether they underestimated or overestimated their weight status.
- Other weight-related information from the World Health Organisation STEPS questionnaire [36] included waist circumference, family history of obesity, weight changes in the last 12 months, and receipt of weight advice from a health-care professional.
- The Beck Cognitive Insight Scale (BCIS), a commonly used and well-validated measure of cognitive insight, was used to assess for objectivity and reflectiveness (higher = better insight) and self-certainty (lower/ less confidence in own beliefs = better insight) [27]
- The Wechsler Abbreviated Scale of Intelligence (WASI) was used to estimate global IQ [37], (to potentially include as covariate).
- The Repeatable Battery for the Assessment of Neuropsychological Status (RBANS), assessed immediate and delayed memory, attention, language, and visuospatial skills [38]. The following tests of Executive Functioning were also administered: The Ruff Figural Fluency Test [39], Stroop colour word test [40], Digit span and Spatial span / Corsi blocks [41] . An estimate of overall cognitive ability was computed by creating z-scores for each RBANS and executive functioning test, which were then added up to create a total neurocognition score.

### Statistical analyses

Chi-square (χ2) tests were used to determine which categorical variables were associated with PBW and ANOVA or Mann-Whitney U tests, as appropriate, were run to determine the continuous variables that were associated with PBW. Significant factors from univariate analyses were entered into a stepwise logistic regression models to identify the factors that were significantly associated with accurate PBW. IBM SPSS version 26 was used to analyse the data with a 2-sided significance level set at 0.05.

## Results

**Fig. 1:**
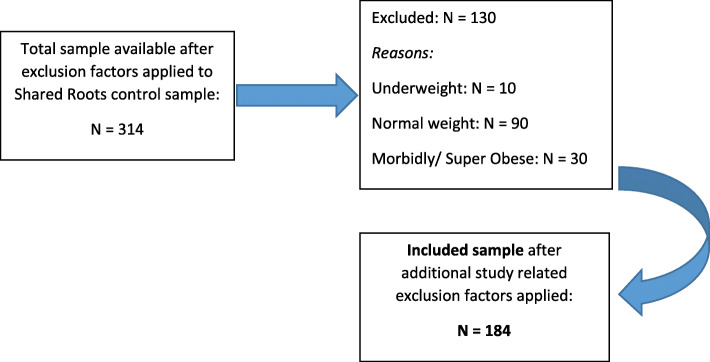
Flow diagram of sample selection

A description of the full and included samples is provided in Table 1. We excluded underweight individuals (the only way they could misperceive their weight was to overestimate it) and normal weight individuals (the only way normal weight individuals could underestimate their weight was to call themselves underweight) from the analysis. We decided not to analyse individuals who overestimated their weight because of the small sample, and few normal weight individuals underestimated their weight). We also excluded morbidly and super morbidly obese individuals (i.e. class III) from further analysis, as none of these individuals perceived themselves to be underweight or of normal weight (Table 2). This left us with a sample size of *N* = 184 (see Fig. 1: Flow chart of sample selection). The full and included samples differed significantly with regard to age (the included sample was older), marital status (more individuals in the included sample were never married) and waist circumference (waist sizes were larger in the included sample).

**Table 1:** Demographic, Weight and Neurocognitive Variables for Full and Included Samples

	***Full sample: N = 314***	***Included sample: N = 184***
**Variable**	**N (Percentage)**	**N (Percentage)**
Gender	Male: 97 (30.9)	Male: 47 (25.5)
	Female: 217 (69.1)	Female: 137 (74.5)
**Education (highest level completed)	< Primary school: 28 (8.9)	< Primary school: 20 (10.9)
	Some Secondary School: 163 (51.9)	Some Secondary School: 100 (54.3)
	Completed Secondary School: 123 (39.2)	Completed Secondary School: 64 (34.8)
Marital Status ^#^	Married/ Cohabitating: 138 (43.9)	Married/ Cohabitating: 52 (28.3)
	Never Married: 119 (37.9)	Never Married: 98 (53.3)
	Divorced/ Separated/	Divorced/ Separated/
	Widowed: 57 (18.2)	Widowed: 34 (18.5)
Employment	Yes: 122 (38.9)	Yes: 63 (48.2)
	No: 192 (61.1)	No: 121 (65.8)
Monthly Income	< R1500: 46 (14.6)	< R1500: 24 (13.6)
	R1500 – R3000: 86 (27.4)	R1500 - R3000: 54 (30.5)
	R3000 - R6000: 77 (24.5)	R 3000 - R6000: 49 (27.7)
	R6000 – R12000: 49 (15.6)	R6000 - R12000: 24 (13.6)
	> R12000: 41 (13.1)	> R12000: 26 (14.7)
Weight Perceived Correctly	Yes: 209 (66.6)	NA
	No: 105 (33.4)	
Underestimated Weight	Yes: 92 (29.3)	NA
	No: 222 (70.7)	
Overestimated Weight	Yes: 13 (4.1)	NA
	No: 301 (95.9)	
**Ever overweight/ obese	Yes: 141 (44.9)	Yes: 105 (57.1)
	No: 173 (55.1)	No: 79 (42.9)
**Weight changes in previous year	Yes – weight increased: 69 (22)	Yes – weight increased: 49 (26.6)
	Yes – weight decreased: 45 (14.3)	Yes – weight decreased: 24 (13.0)
	No: 200 (63.7)	No: 111 (60.3)
**Family member overweight	Yes: 118 (37.6)	Yes: 76 (41.3)
	No: 190 (60.5)	No: 108 (58.7)
**Told to maintain healthy weight by health-care professional	Yes: 119 (37.9)	Yes: 77 (41.8)
	No: 195 (62.1)	No: 107 (58.2)
**Variable**	**Mean (SD)**	**Mean (SD)**
*Age in years ^#^	45.82 (15.62)	49.34 (14.97)
	Range: 18.06–81.12	Range: 18.25–81.12
Waist Circumference ^#^	91.13 (15.77)	95.37 (10.47)
**Body Mass Index	29.65 (7.68)	31.37 (3.95)
Self-Reflectiveness	3.41 (5.32)	3.32 (5.65)
**Self- Certainty	9.75 (3.39)	9.65 (3.42)
*BCIS Total	13.17 (4.94)	12.97 (5. 51)
**RBANS Immediate Memory	44.58 (7.87)	44.01 (7.85)
RBANS Visuospatial	32.25 (4.64)	32.27 (4.12)
*RBANS Language	27.58 (5.67)	27.61 (4.52)
**RBANS Attention	47.39 (12.91)	46.20 (12.77)
**RBANS Delayed Recall	47.65 (7.54)	47.23 (7.43)
**RBANS Total	199.35 (29.11)	197.11 (28.44)
**Global IQ (WASI)	67.33 (15.61)	68.61 (13. 54)
RFFT Error Ratio	0.17 (0.21)	.16 (0.20)
Stroop Interference	−1.58 (7.84)	−2.40 (8.54)
*Spatial Span Backwards	6.07 (2.18)	5.92 (2.14)
Digit Span Backwards	5.29 (2.21)	5.25 (2.34)
Total Executive Function (z-score)	0.03 (2.47)	−0.04 (2.48)
Total Neurocognition (z-score)	−0.03 (3.13)	− 0.27 (3.10)

Numbers may not add up to 100% due to missing values

*BCIS:* Beck Cognitive Insight Scale, *IQ:* Intelligence Quotient, *RBANS:* Repeatable Battery for the Assessment of Neuropsychological Status, *WASI:* Wechsler Abbreviated Scale of Intelligence

# Full and included samples differed significantly with regard to age, marital status and waist circumference

*: relationship with correct perception of body weight *in included sample* - *p* ≤ 0.10

**: relationship with correct perception of body weight *in included sample* - *p* ≤ 0.05

**Table 2:** BMI Classification and Weight Perception Numbers

	Weight perception		
BMI Classification	Underweight	Normal Weight	Overweight
Underweight	4	6	0
Normal	18	65	7
Overweight	5	44	29
Obese Class I	0	20	47
Obese Class II	0	5	34
Obese Class III	0	0	30

In univariate analysis the following variables were associated with incorrect perceptions of body weight (*p* ≤ 0.05): education; BMI; reported weight loss/ gain in the previous year, reported ever being obese or having a family member (blood relative) who was obese; being told to lose weight or maintain a healthy body weight by a health-care professional; self-certainty (on the BCIS); global IQ (assessed by the WASI); and the RBANS total score (memory, attention and delayed recall scores contributed to this). These variables, excluding ever being obese and global IQ, were entered into a logistic regression. For the variable ‘ever obese’, the same individuals who responded positively to this acknowledged being currently overweight while the variable ‘global IQ’ was significantly correlated with the RBANS score. We also included age, which trended towards significance (*p* = 0.061), in the model. C*ognitive variables* (self-certainty; RBANS Total score) were entered in the first step, *weight-related variables* (BMI, reporting that one had gained weight in the previous year, that one had had blood relative who was obese, that one was told to lose weight or maintain a healthy body weight by a health-care professional) in step two and d*emographic variables* (age, education) in the final step.

*Cognitive variables* alone explained 7.1% of variation (Nagelkerke R^2^), and correctly identified 64.7% of individuals who correctly and incorrectly perceived their weight. This is 4.9% higher than when the constant alone was included in the model. When *weight-related variables* were added to the model, 50.5% of variation was explained and 78.8% of individuals who correctly and incorrectly perceived their weight were correctly identified. The final logistic regression model, which include the *demographic variables* and, explained 52.3% of variation in accurate perception of body weight, correctly classified 79.3% of individuals who correctly and incorrectly perceiving their weight, and was significant (χ2 (10, *N* = 184) = 90.137, *p* ≤ 0.01). Table 3 provides a summary of each variable in the equation whilst controlling for the other variables. The RBANS total score, having overweight blood relatives, age and education were not significant when other variables in the equation were controlled for. Self-certainty, BMI, reported weight change and having being told to lose/ maintain a healthy weight by a health care professional and were significant in the final equation. Individuals with greater self-certainty and those with lower BMI were less likely to perceive their weight correctly; while those who reported that they had gained weight in the previous year or were told by a health-care professional to lose or maintain a healthy weight were more likely to perceive their weight correctly. For example, reporting that one was told to lose or maintain a healthy weight by a healthcare professional resulted in an increase of 0.985 in the log-odds of the dependent variable (correct perception of weight), holding all other independent variables constant.

**Table 3:** Hierarchical Regression of Factors Associated with Perception of Body Weight

Predictor	Β (coefficient for the constant)	SE β	Wald’s χ2	df	*p* (Significance)	*e*^β^ (Odds ratio)	95% C.I.for EXP(B)	
							Lower	Upper
RBANS: total score	−0.015	0.010	02.110	1	> 0.05	0.985	0.966	1.005
BCIS: self-certainty total score	0.163	0.062	6.944	1	≤0. 01**	1.177	1.043	1.328
BMI	−0.366	0.068	29.172	1	≤0.001**	0.694	0.607	0.792
Weight changes			14.725	2	≤0.001**			
Weight changes (1)	1.205	0.684	3.102	1	> 0.05	3.338	0.873	12.761
Weight changes (2)	−1.633	0.534	9.360	1	≤0. 01**	0.195	0.069	0.556
Overweight blood relatives	−0.482	0.423	1.299	1	> 0.05	0.617	0.269	1.415
Maintain a healthy body weight or lose weight	0.985	0.460	4.591	1	≤0.05*	2.678	1.088	6.591
Age	0.006	0.016	0.124	1	> 0.05	1.006	0.974	1.038
HLOE			3.552	2	> 0.05			
HLOE (1)	−0.874	0.684	1.630	1	> 0.05	0.417	0.109	1.596
HLOE (2)	−1.517	0.816	3.455	1	> 0.05	0.219	0.044	1.086
Constant	12.747	3.385	14.179	1	≤0.001**	343,400.256		

*BCIS:* Beck Cognitive Insight Scale, *BMI:* Body mass index, *HLOE:* Highest level of education; RBANS: Repeatable Battery for the Assessment of Neuropsychological Status

*HLOE* Reference category = 0 = Primary school or less, 1 = Some secondary school, 2 = Completed secondary school

Weight changes: Reference category = 0 = No significant weight changes, 1 = Weight decreased, 2 = Weight increased

*: relationship with correct perception of body weight - *p* ≤ 0. 05

**: relationship with correct perception of body weight - *p* ≤ 0.01

Note: Supplementary Table 1 compares these results to regression results when those who misperceived their weight to be higher were included in the analysis

## Discussion

This is the first study, to our knowledge, to demonstrate a strong association between cognitive insight, rather than cognitive function, on PBW. Over one third of individuals in the study misperceived their weight, with the majority underestimating their weight. Weight underestimation was also associated with greater self-certainty. The prevalence of misperception of body weight in this sample is lower than other studies conducted in sub-Saharan Africa [7, 42, 43], as well as lower than that found in other LMICs [44, 45]. It has been suggested that cultural preferences for obesity, and limited opportunities to measure actual weight may contribute to misperceptions about body weight [42]. Given that recruitment was targeted at individuals at risk for cardiovascular disease, many individuals in our study might have already been aware of their weight status.

We aimed to determine whether better cognitive functioning and better cognitive insight were associated with PBW. In univariate analysis, those with a higher global IQ and neurocognition score (as assessed on the RBANS) were more likely to perceive their weight correctly. This relationship did not hold once other variables were accounted for. Despite the association between obesity and impaired cognitive performance [46, 47], this relationship does not seem to extend to correct perception of body weight.

In both univariate and multivariate analyses, those with high self-certainty were more likely to underestimate their weight. Confidence and satisfaction with oneself is likely to allow individuals to view themselves in a more positive light as demonstrated by the relationship between self-certainty and self-esteem [48]. Given that perceptions of obesity and overweight may have cultural determinants this may reflect a cultural belief that overweight/ obesity is ‘normal’ and desirable [49]. For instance, a qualitative study conducted in a black South African population found that most of the obese and overweight women perceived themselves to weigh less than they did, and expressed satisfaction with their body sizes [50]. Additionally, both overweight and normal weight women perceived large body sizes as attractive and ‘normal’ [50]. As such, these individuals may be less responsive to other external cues (e.g. persuasion,) of their current weight status, and may require more direct feedback.

We also aimed to identify *weight-related* and *sociodemographic* factors associated with correct perception of body weight. In univariate analysis the presence of an obese family member and higher level of education were significantly associated with correct PBW, while age trended towards significance. These variables were no longer significant when other variables were accounted for. This is in contrast to other studies, including those in LMIC that have found demographic factors such as SES, age and gender to be related to PBW [42, 43, 51]. BMI, reporting that one had gained weight in the previous year, ever being obese or being previously told to lose weight or maintain a healthy body weight by a healthcare professional remained significant in the multivariate model.

Those with a lower BMI were more likely to underestimate their weight. Considering that this sample comprised only overweight and obese participants, this means the closer one is to a normal BMI, the more likely they are to think they are normal – as weight increases the less likely participants are to think they are normal and/or weight gain becomes harder to deny. A Norwegian study of overweight individuals had similar results- finding that a higher BMI was protective against weight underestimation [15]. However, as our categories were limited to three groups (participants could only select whether they perceived themselves to be underweight, normal weight or overweight) this decreased our sensitivity to pick up whether individuals with very high BMIs were also underestimating their weight – these individuals, for instance, might think they are overweight when they are in fact obese.

Those who reported that they had gained weight were less likely to underestimate their weight. This may relate to perceptions of oneself- if in denial of being overweight an individual may also be in denial of having gained weight. Alternatively, it may be that, having gained weight, these individuals are more aware of their current weight status. A study that assessed parental perception of child overweight two years later, for example, found that perception of overweight seemed mainly to reflect an awareness of an already rising trajectory [52].

Having a healthcare worker tell one to lose weight/ maintain a healthy body weight decreased the likelihood of individuals underestimating their weight, when other factors were controlled for. This stands to reason, given that this would have raised participants awareness of their body weight, and aligns with other research [53]. This could also suggest that the advice of healthcare professionals is more effective than other forms of social influence and highlights the relevance of physician informed strategies in reducing weight-related morbidity. Nonetheless, accurate perception of weight does not necessarily relate to changes in behaviour [52, 54].

Some limitations should be considered. First, in examining the relationship between BMI classification and perception of weight, categories of underweight, overweight and normal weight may have been too restrictive to determine the accuracy of weight perception in morbidly and super morbidly obese individuals, as their weight was too far out of the normal range. We therefore excluded these individuals from our analysis. Second, we further excluded individuals who overestimated their weight as different factors have been found to be associated with overestimating than underestimating weight [55], and there were too few individuals who overestimated their weight to analyse this group separately. Third, normal weight individuals were also excluded as the only way they could misperceive their weight was to say they were underweight, and very few participants did so. It is also likely that different factors come into play here. Fourth, this was a cross-sectional study limiting inferences about directionality. Longitudinal studies will allow more accurate determination of the association between weight perception and later physical and mental health behaviours and health outcomes. A review of the association between obesity and cognitive function, for example, found longitudinal evidence that lower childhood intelligence leads to obesity and weight gain in adulthood, with no credible evidence to the contrary [56]. Fifth, the BCIS is not the ideal measure to evaluate insight in non-psychotic patients. Future studies should preferably use an obesity related insight measure such as The Obesity Awareness and Insight Scale [57]. Finally, as participants were recruited using purposive sampling they may not represent a random population and generalisability may be limited.

## Conclusion

Despite these limitations, these findings suggest that some aspects of cognitive insight (self-certainty) but not cognitive functioning are associated with perception of body weight in this sample. Furthermore, weight-related variables, such as BMI, reporting that one had gained weight in the previous year, ever being obese, or being previously told to lose weight or maintain a healthy body weight by a healthcare professional, were associated with perceptions of body weight in this sample, while demographic variables were not. Understanding factors that contribute to the correct perception of one’s weight is important in identifying appropriate public health interventions such as Cardiovascular Disease prevention programs. Clinically, our results may indicate that healthcare providers need to be more vigilant in identifying those who are overweight and counsel these individuals regarding health and lifestyle modification, for example. Given that misperception of one’s weight (under- and over-) can influence physical and mental health, such interventions may address the burden of associated non-communicable diseases in this population. Even so, weight underestimation may also be protective of one’s mental health. As such, the potential negative effects of correcting weight misperceptions need to be considered when implementing lifestyle and behavioural interventions.

## Supplementary Information

**Additional file 1: Supplementary Table**: Hierarchical Regression of Factors Associated with Perception of Body Weight in (A) ‘Included’ sample and (B) ‘Included’ sample and those who misperceived their weight to be higher.

## Data Availability

Data will be made available upon reasonable request. Requests may be sent to Sharain Suliman: sharain@sun.ac.za.

## Abbreviations

BCIS: Beck Cognitive Insight Scale
BMI: Body Mass Index
cm: Centimetres
IQ: Intelligence Quotient
kg: Kilograms
LMIC: Low- and middle-income countries
MINI: MINI International Neuropsychiatric Interview
PBW: Perceived body weight
RBANS: Repeatable Battery for the Assessment of Neuropsychological Status
SES: Socio-economic status
WASI: Wechsler Abbreviated Scale of Intelligence
