# Supplementary material for: Cognitive insight is associated with perceived body weight in overweight and obese adults
Source: BMC Public Health. 2021 Mar 19;21:534. doi: 10.1186/s12889-021-10559-5 (PMC7976704; doi:10.1186/s12889-021-10559-5)
Supplement: Supplementary file 1 — Additional file 1: Supplementary Table: Hierarchical Regression of Factors Associated with Perception of Body Weight in (A) ‘Included’ sample and (B) ‘Included’ sample and those who misperceived their weight to be higher. [file 12889_2021_10559_MOESM1_ESM.docx]

Supplementary Table: Hierarchical Regression of Factors Associated with Perception of Body Weight in (A) ‘Included’ sample and (B) ‘Included’ sample and those who misperceived their weight to be higher

| **Sample:** | **A** | **B** |
| --- | --- | --- |
| **Variables** | (χ2 (10, N=184) = 90.137, p≤0.01) | (χ2 (9, N=195) = 91.428, p≤0.01) |
| **Cognitive** | Nagelkerke R^2^: 7.1% | Nagelkerke R^2^: 4.7% |
|  | Correctly identified: 64.7% (4.9% higher than constant alone) | Correctly identified: 62.1% (29.9% higher than constant alone) |
| **Significant variables** | Self-certainty | Self-certainty |
| **Weight related** | Nagelkerke R^2^: 50.5% | Nagelkerke R^2^: 48.6% |
|  | Correctly identified: 78.8% | Correctly identified: 79.5% |
| **Significant variables** | BMI; Weight changes; Maintain a healthy body weight or lose weight | BMI; Maintain a healthy body weight or lose weight (p<0.10) |
| **Demographic** | Nagelkerke R^2^: 52.3% | Nagelkerke R^2^: 50.2% |
|  | Correctly identified: 79.3% | Correctly identified: 80.5% |
| **Significant variables** | - | - |

A: Included sample

B: Included sample and those who misperceived their weight to be higher
